# The Impact of Estrogen on Stromal Elements in the Proximal Airway in Idiopathic Subglottic Stenosis

**DOI:** 10.1002/lary.70216

**Published:** 2025-11-14

**Authors:** Emily L. Mace, Evan Clark, Edward Talatala, Maxim Litvak, Matthew A. Buendia, Yash A. Choksi, Hongmei Wu, Yueli Zhang, Alexander Hillel, Sam Collins, Quanhu Sheng, Jing Yang, Jason Park, Alexander Gelbard

**Affiliations:** ^1^ Department of Otolaryngology‐Head & Neck Surgery Vanderbilt University Medical Center Nashville Tennessee USA; ^2^ Department of Pediatrics, Division of Gastroenterology, Hepatology, and Nutrition Vanderbilt University Medical Center Nashville Tennessee USA; ^3^ Department of Medicine, Division of Gastroenterology, Hepatology and Nutrition Vanderbilt University Medical Center Nashville Tennessee USA; ^4^ Department of Medicine Veteran's Affairs Hospital, Tennessee Valley Health System Nashville Tennessee USA; ^5^ Department of Otolaryngology‐Head & Neck Surgery Johns Hopkins University School of Medicine Baltimore Maryland USA; ^6^ Department of Biostatistics Vanderbilt University Medical Center Nashville Tennessee USA

**Keywords:** estrogen, fibrosis, idiopathic subglottic stenosis

## Abstract

**Objectives:**

Idiopathic subglottic stenosis (iSGS) is an unexplained fibrosis of the proximal airway that predominantly impacts Caucasian women. Clinically, premenopausal patients suffer from higher recurrence rates, potentially implicating ovarian hormone 17B‐estradiol (E2) in disease pathogenesis. Despite its relationship with disease phenotype and severity, the mechanistic role of E2 in subglottic fibrosis has not been investigated.

**Methods:**

Primary human cell lines derived from the airway scar of iSGS patients were utilized for in vitro study to assess the impact of E2 on proximal airway cells (fibroblasts, epithelial cells, and endothelial cells). Alterations in RNA and extracellular matrix protein expression were assessed in fibroblasts (*n* = 5 primary cell lines) following E2 exposure. The impact of E2 on epithelial barrier function was assessed via transepithelial electrical resistance measurement (TEER) in iSGS‐patient‐derived epithelial primary cell lines (*n* = 3) grown via air–liquid interface (ALI) culture. The impact of E2 on iSGS‐patient‐derived endothelial lines was assessed using a Matrigel‐based tube formation assay.

**Results:**

There were no significant transcriptional changes induced in fibroblasts after exposure to E2 when compared to controls, nor was a difference in collagen production observed after E2 exposure. There was no significant difference in TEER measurements in airway epithelial cells grown at ALI following E2 exposure. Endothelial cells showed a significant increase in tube formation following E2 exposure.

**Conclusion:**

In vitro models suggest E2 may have limited direct impact on fibroblasts and epithelial cells in iSGS. Instead, estrogen acts directly on airway endothelial cells to drive vascular remodeling, potentially contributing to mucosal fibrosis.

**Level of Evidence:**

NA.

## 1 Introduction

Idiopathic subglottic stenosis (iSGS) is a localized fibrosis of the proximal airway mucosa that occurs almost exclusively in adult Caucasian women [1, 2, 3]. Given the extreme gender bias in affected patients, female sex is hypothesized to play a critical role in iSGS development. Yet, the mechanistic role of ovarian hormones in proximal airway remodeling is poorly understood and dramatically understudied. Illuminating the biological basis for the female predominance of iSGS may provide critical insights into disease pathogenesis and guide the development of new therapies [1].

iSGS is characterized by an accumulation of fibrous connective tissue, including collagen and fibronectin, in the setting of persistent mucosal inflammation [4, 5]. Previous mechanistic studies suggest that iSGS patients possess defects in epithelial barrier function that contribute to dysregulated immune activation and promote fibrotic mucosal remodeling [6, 7]. Indirect evidence also supports a role for estrogen in proximal airway fibrosis. The predominance of female sex in airway fibrosis is conserved across several airway pathologies, suggesting the airway is particularly susceptible to the effects of sex hormones. Although women and men are equally affected by systemic Granulomatosis with polyangiitis (GPA: 53% vs. 47%), female GPA patients have a higher rate of subglottic stenosis (72% vs. 28%) [8]. The same female gender bias is seen in subglottic stenosis arising after prolonged intubation (PIPS). Analysis of a large inpatient dataset revealed female gender doubled the risk for PIPS (OR = 1.96, 95% CI = 1.58–2.44) [9]. Interestingly, the PIPS female gender bias disappeared after age 50, around the time of menopause, suggesting E2 may have a critical role in PIPS development. The female predominance of iSGS, GPA, and PIPS patients reinforces the critical role E2 has in subglottic fibrosis after injury.

In premenopausal women, the major circulating estrogen is 17β‐estradiol (E2) [10, 11], which signals through classical estrogen receptors ER‐α and ER‐β [12, 13]. Additionally, E2 can also signal via alternate pathways such as G protein‐coupled estrogen membrane receptor (GPER1) [14]. Published data from our group and others have localized these receptors within the cell subsets of iSGS airway scar [15, 16, 17]. While this prior work supports a role of sex hormone dysregulation among the stromal elements within the proximal airway, mechanistic study in iSGS is lacking. Using samples from iSGS patients, this study explored the impact of estrogen on the epithelial cells, fibroblasts, and endothelial cells in iSGS airway scar.

## 2 Materials and Methods

This study was performed in accordance with the Declaration of Helsinki, Good Clinical Practice. This study was approved by the Institutional Review Board at Vanderbilt University Medical Center, Nashville, Tennessee #140429.

### 2.1 Tissue Sampling and Cell Culture

Human tissue samples were taken from patients with an established diagnosis of iSGS with previously described clinical and serologic criteria [18]. Samples were obtained from subglottic scar intraoperatively from premenopausal female patients who had a normal course of disease severity as previously described [6]. Tissue samples were immediately placed in sterile room temperature saline and transferred to the lab for tissue digestion after acquisition from the patient during direct laryngoscopy with cup forceps. Samples were then placed in an enzymatic cocktail 0.1% collagenase I, DNase 500 μg/mL in 5 mL of 10% FBS DMEM medium at 37°C for 40 min with constant agitation.

For isolation of scar endothelial cells, Invitrogen Dynabeads CD31 endothelial cell kit (Invitrogen by Thermo Fisher Scientific cat#11155D) was used to select endothelial cells. Selected endothelial cells were plated in a 25 cm^2^ flask in a vascular cell basal medium (ATCC PCS‐100‐030). Cells were then incubated at 37°C, 5% CO_2_ for 3–5 days until 80%–90% confluence. Endothelial cells were reselected using Dynabeads CD31 endothelial cell kit, expanding cells into 75 cm^2^ flask with a cell density of 1.5 × 10^6^ in a vascular cell basal medium. At least two additional endothelial cell selections were performed before confirmation by flow cytometry with antibody CD31 Pacific Blue (BioLegend cat#303114).

For isolation of scar fibroblast cells, cells were isolated and seeded onto 24‐well culture plates (Falcon, Tewksbury, Massachusetts) at 100,000 cells per well and cultured in standard media as previously described. All proliferation experiments were conducted in triplicate for each biological replicate [19, 20].

For isolation of scar epithelial cells, cells were differentiated in vitro in PneumaCult‐ALI (STEMCELL Technologies Inc., Seattle, WA) medium for 4 weeks as previously described [21]. Cultures reaching 80% confluency were seeded onto human collagen type IV‐coated (Sigma, St. Louis, MO) transwell inserts (0.33‐cm^2^ polyester, 0.4‐μm pore size; Corning Costar, Tewksbury, MA) at a density of 3 × 10^5^ cells per well with apical and basolateral PneumaCult‐Ex Plus medium (STEMCELL Technologies Inc.). Once 95% confluency was achieved, cells were differentiated in PneumaCult‐ALI (STEMCELL Technologies) for 4 weeks [21, 22, 23, 24]. Primary human healthy proximal airway epithelial cells were obtained from Lonza Bioscience and cultured per manufacturer instructions.

### 2.2 Transepithelial Electrical Resistance

To assess the impact of E2 on epithelial barrier integrity, transepithelial electrical resistance (TEER) was measured after exposure to E2 [25]. Since estrogen exposure is believed to impact biological function largely through transcriptional regulation, experiments were designed to evaluate changes on that timescale. Therefore, TEER values were measured at the time scale of weeks (two, three, and four) for sufficient exposure. E2 levels in these experiments were chosen to reflect the biologic range of the hormone found in premenopausal females and previously established for in vitro experiments [26, 27]. Primary epithelial cells were seeded at a density of 50,000/100 μL on collagen‐coated inserts in 24‐well plates and cultured at 37°C and 5% CO_2_. The entire well (both apical and basal chambers) was filled with media. Once cells reached confluence and generated TEER values greater than 200 Ω cm^2^, air–liquid interface (ALI) was started with the removal of media from the apical chamber. Media was transitioned to a combination of differentiation and standard media (mixed at an 80%–20% ratio). This mixture was then distributed only to the basal chamber to promote differentiation of a ciliated airway epithelial layer. After 1–2 days, media was then transitioned to 100% differentiation media to the basal layer. Seven days after the initiation of ALI, exposure to E2 was started. Basal wells were exposed to either control or E2 (ranging from 2 pM to 20 nM). TEER was measured with the Millicell ERS‐2 Epithelial Voltohmmeter at 21 days after ALI initiation. Throughout this time, media was changed gently every 2 days along with E2 treatments.

### 2.3 Flow Cytometry

Flow cytometry was used to assess the presence of estrogen and progesterone receptors in healthy airway epithelium, and the epithelium and endothelium from iSGS tissue samples. Cells were stained with viability dye and Fc‐Block for 10 min. Cells were then fixed following the Invitrogen Intra Cellular (IC) Fixation kit and then washed once with permeabilization (perm) buffer. Antibodies to the estrogen and progesterone receptors were applied for 30 min and then washed twice with Perm wash. Cells were then resuspended with 200 μL of perm washing buffer for data acquisition. Flow cytometry was employed to quantify fibroblast activity via measurement of collagen 1A levels as previously described [19]. All flow cytometry experiments were acquired with an LSR‐II flow cytometer (BD Biosciences) and analyzed using FlowJo (FlowJo LLC, Ashland, OR). A minimum of 200,000 events was acquired for each sample.

### 2.4 RNA Sequencing

Bulk RNA sequencing was used to measure differential gene expression when fibroblasts were exposed to estrogen (or progesterone). iSGS airway scar fibroblasts were grown in 10 mm cell culture dishes with Dulbecco's Modified Eagle Medium (DMEM) until 90% confluence. They were then transferred to 12‐well cell culture dishes with low serum (DMEM + 0.4% FBS) for 24 h. Medium was then changed to complete medium (DMEM with 10% FBS and 1% penicillin/streptomycin). E2 and progesterone (PG) were added to separate wells to obtain a concentration of E2 at 2 × 10^−12^ (2 pM) and progesterone at 2 ng/mL. Cells were incubated for 6 h at 37°C at 5% CO_2_. Cells were then harvested for RNA extraction with the RNeasy Plus Mini Kit per kit instructions. Sample RNA quality control was validated using fluorometry Qubit and integrity by BioAnalyzer. The NEBNext Poly(A) selection kit was used for Library Preparation. All samples were sequenced at Paired‐End 150 bp on the Illumina NovaSeq 6000 targeting an average of 50 M reads per sample.

Reads were trimmed to remove adapter sequences using Cutadapt (v2.10) [28] and aligned to the Gencode GRCh38.p13 genome using STAR (v2.7.8a) [29]. Gencode v38 gene annotations were provided to STAR to improve the accuracy of mapping. Quality control on both raw reads and adaptor‐trimmed reads was performed using FastQC (v0.11.9). FeatureCounts (v2.0.2) [30] was used to count the number of mapped reads to each gene. Heatmap3 [31] was used for cluster analysis and visualization. Significantly differential expressed genes with absolute fold change ≥ 2 and false discovery rate adjusted *p* value ≤ 0.05 were detected by DESeq2 (v1.30.1) [32]. Gene set enrichment analysis was performed using GSEA package (v4.1.0) [33] on database (v7.4).

### 2.5 Angiogenesis Measurements

Endothelial cell lines from iSGS patients were co‐cultured with fibroblasts per established protocol [34] with the following modifications. On day 3, media were changed to either standard endothelial basal media (EBM‐2: Lonza, the negative control group), standard media +2 ng/mL VEGF (positive control), or standard media +5 nM E2 (experimental “E2” group). Fourteen wells for each group, as well as one well for controls containing either fibroblasts only or endothelial cells only for each media composition, were co‐cultured for 7 days with media changed every 2 days.

Following co‐culture, cells were washed twice with phosphate buffered saline (PBS) and fixed with ice‐cold 70% ethanol for 30 min. Wells were then washed twice with PBS and subsequently stained with anti‐CD31 fluorophore (AF647)‐conjugated mouse antibody (BioLegend) in PBLEC staining solution. After staining, cultures were washed twice with PBS. A 4′,6‐diamidino‐2‐phenylindole (DAPI) counterstain was then applied as per Springer Angiogenesis protocol. Images were taken with a Nikon fluorescent microscope and were analyzed using ImageJ Angiogenesis Analyzer, a validated automated program for assessing multiple measures of angiogenesis via endothelial tube formation assay (ETFA) [35]. The outer 30% area of the plates was cropped prior to analysis due to distortion when captured on microscopy caused by perpendicular growth of cells along plate walls. The analyzed measures include but are not limited to total length, total branching length, nodes, and total segment length.

### 2.6 Statistical Analysis

Significance was set to 5% (alpha = 0.05) in all analyses. Normal distribution of variables was tested with the Shapiro–Wilk test. Differences between *x* and *y* groups were determined with the Kruskal–Wallis and Mann–Whitney tests for normal and nonnormal distributions, respectively. All statistical analyses were performed with Prism 10.0 software (GraphPad Software Inc., La Jolla, California).

## 3 Results

**FIGURE 1:**
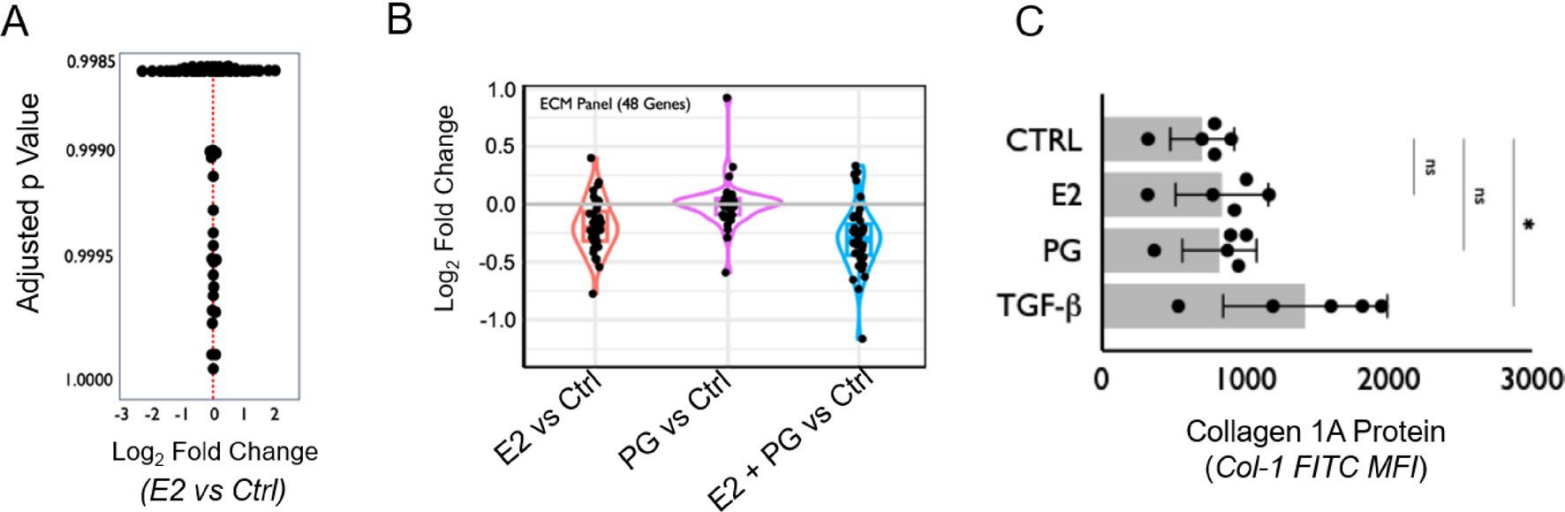
(A) Transcriptional analysis of iSGS fibroblast cell lines (*n* = 5) following 72 h of E2 exposure. No significant differences in RNA expression detected. (B) Analysis of transcriptional changes in a curated panel of 48 gene extracellular matrix (ECM) genes in iSGS fibroblast cell lines (*n* = 5) following 72 h of exposure to E2, PG, or E2 + PG. No significant difference in ECM panel when exposed to E2, PG, or E2 and PG. (C) Collagen 1A (Col1A) protein levels in iSGS fibroblast cell lines (*n* = 5) following 72 h of E2, PG, or TGF‐B exposure measured via flow cytometry. No significant difference in Col1A protein expression detected after E2 or PG exposure. [Color figure can be viewed in the online issue, which is available at www.laryngoscope.com]

To assess the impact of E2 on stromal fibroblasts in iSGS proximal airway stenosis, we first exposed primary fibroblast cell lines derived from airway scar from five iSGS patients to E2 (2 pM), PG (2 ng/mL), or E2 and PG together or no treatment for 6 h. Bulk RNA sequencing (RNAseq) compared cell transcriptional changes induced by E2 in vitro. Surprisingly, there was no significant difference in the expression of RNA after E2 exposure (Figure 1A, *p* > 0.1). Similarly, there was no significant difference in RNA expression after PG or E2 + PG (data not shown). Next, we analyzed a curated panel of 48 genes associated with extracellular matrix (ECM) genes (Table S1) for transcriptional changes after E2, PG, or E2 + PG. There was no significant difference in the expression of any genes in the ECM panel in any of the conditions (Figure 1B). To confirm that E2 did not alter ECM protein production, we exposed iSGS fibroblast cell lines (*n* = 5) to E2 (2 nM), PG (2 nM), or positive control TGF‐β (20 ng/mL) for 36 h. Flow cytometry demonstrated no significant difference in collagen 1A production when exposed to E2 or PG when compared to untreated controls.

Next, we explored the functional impact of E2 on proximal airway epithelial cells.

**FIGURE 2:**
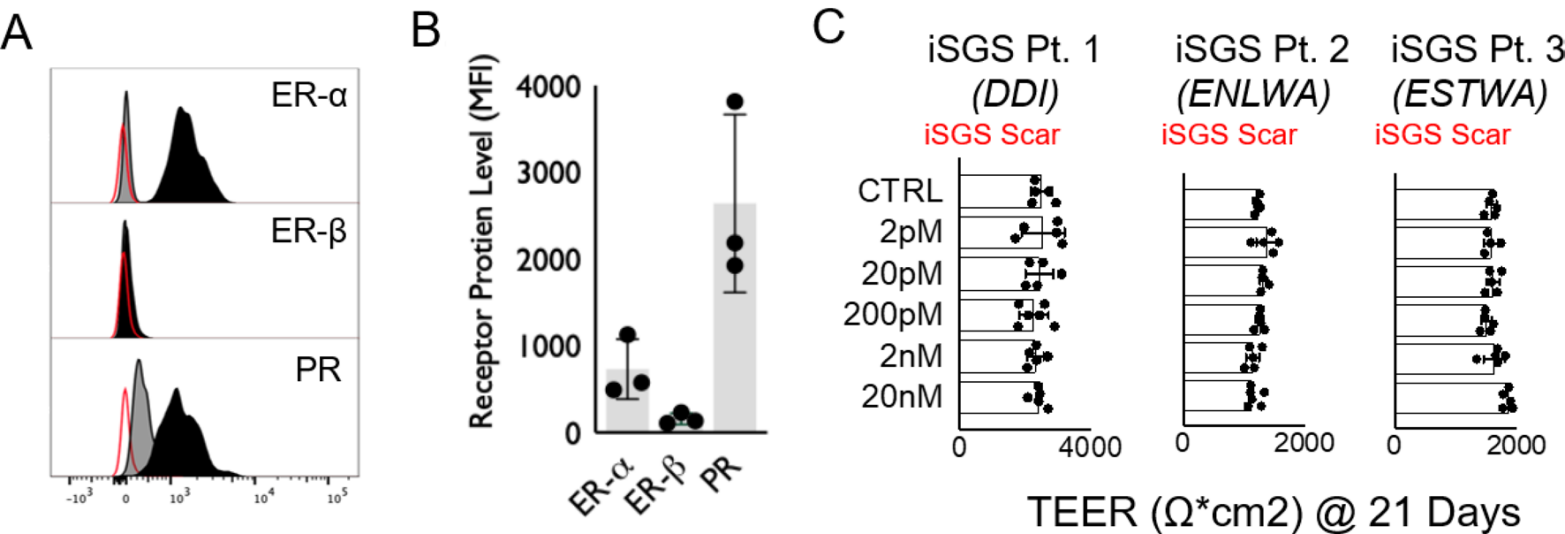
(A) Presence of estrogen and progesterone receptors in healthy proximal airway cells (Lonza). Red line represents unstained cells, gray line denotes isotype control, and black line anti‐receptor antibody. (B) Quantification of the Mean Florescent Intensity (MFI) of flow cytometry demonstrates presence of ER‐α, ER‐β, and PR in three iSGS scar samples. (C) No significant difference in TEER in iSGS scar after 21 days. [Color figure can be viewed in the online issue, which is available at www.laryngoscope.com]

We first assessed the expression of estrogen and progesterone receptors in healthy proximal airway epithelial cell lines. These cells were found to express ER‐α and progesterone receptors but demonstrate negligible levels of ER‐β, shown in Figure 2A. Similar results were found when assessing receptor levels in iSGS epithelial lines, shown in Figure 2B. To understand the impact of E2 on proximal airway epithelial barrier function, we utilized 3 biologically distinct iSGS epithelial cell lines grown in ALI. ALI cultures were exposed to varying concentrations of E2 (2 pM–20 nM), and barrier integrity was assessed via Transepithelial electrical resistance (TEER). There were no significant changes in TEER measurements observed in any sample across any levels of E2 exposure.

**FIGURE 3:**
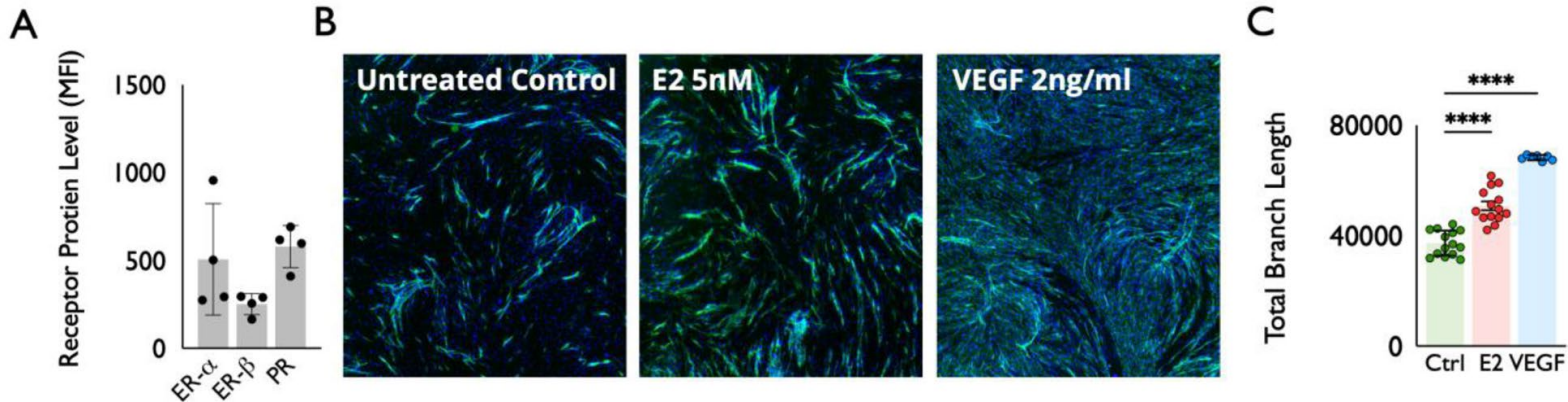
(A) Flow cytometry demonstrates presence of ER‐alpha, ER‐beta, and progesterone receptors in endothelial cells in single cell suspensions derived from iSGS airway scar (*n* = 4). (B) iSGS endothelial cell line grown in co‐culture with iSGS fibroblasts either left untreated, exposed to 2 ng/mL E2 or exposed to 2 ng/mL VEGF. Green anti‐CD31 highlights endothelial cells, DAPI as a background nuclear stain in blue. (C) Quantification of total branch length of endothelial cell lines after culture demonstrate significant increase in total branch length after E2 (*p* < 0.01) and VEGF (*p* < 0.01) exposures compared to untreated control. [Color figure can be viewed in the online issue, which is available at www.laryngoscope.com]

Finally, we explored the functional impact of E2 on proximal airway endothelial cells. We utilized flow cytometry to confirm ER‐α, ER‐β, and progesterone receptors' expression in endothelial cells in single cell suspensions derived from 4 iSGS scar samples, as shown in Figure 3A. We then explored the functional impact of E2 on proximal airway endothelial cells using established angiogenesis assays. Treatment with E2 produced a qualitative increase in angiogenesis, as seen with increased IF staining compared to controls in Figure 3B. Multiple measures of angiogenesis significantly increased with exposure to E2, including total branch length, as demonstrated in Figure 3C (*p* < 0.01). However, this response was not as dramatic in comparison to the positive control, VEGF, in both Figure 3B (*p* < 0.01) and Figure 3C (*p* < 0.01).

## 4 Discussion

These findings demonstrate that E2 increases vessel formation in endothelial cells derived from airway scar in iSGS patients. In contrast, our results demonstrate little effect of E2 on the phenotype or function of iSGS fibroblasts and epithelial cells in vitro.

The proximal airway appears particularly sensitive to the effects of E2. The female predominance of iSGS, GPA, and PIPS patients reinforces the critical role E2 has in subglottic mucosal remodeling after injury. Strong epidemiological evidence also suggests E2 impacts the prevalence and severity of several airway diseases. Severe *asthma* prevalence is increased in women only after puberty [36, 37, 38] but equalizes with men at menopause [39]. While the incidence of Cystic Fibrosis (CF) is not sex linked [33, 40], females with CF exhibit more rapid and diffuse lung disease [39, 41], which are directly associated with E2 levels [42]. Elevated E2 is also the strongest epidemiological risk factor for pulmonary arterial hypertension (PAH) and estrogen antagonism shows promise as a novel therapeutic [16, 17].

These results suggest that in the proximal airway stroma, E2 primarily acts on endothelial cells. Yet, the relationship between E2‐induced angiogenesis and upregulated tissue fibrosis in iSGS remains undefined. Prior work in IPF suggests abnormal endothelial cells and angiogenesis are integral to the development of fibrotic parenchymal remodeling [43, 44]. Human data in pulmonary fibrosis confirms highly abnormal endothelial cells in regions of tissue fibrosis with excessive collagen and elastin surrounding these cells. Additionally, in vitro assays utilizing primary endothelial cell lines derived from IPF patients support dysfunctional endothelial barrier function [45].

The proposed role of angiogenesis in fibroblast activity and fibrosis is not limited to the airway. There has been a significant connection between angiogenesis and hepatic fibrosis, with a positive correlation between the degree of fibrosis and angiogenesis activity. This process seems to work synergistically, with local inflammation resulting in increased vascular permeability, which aids in the development of ECM and fibrosis. This ECM then serves as a scaffolding for endothelial cell migration [46, 47]. It is possible that angiogenesis and fibrosis within the proximal airway synergizes in a similar way. E2 has previously been shown to increase endothelial vascular permeability in human umbilical vein endothelial cells [48]. In iSGS airway scar, there is a significant increase in CD8+ T cell number [49, 50], but how E2 shapes the observed increase in specifically CD8+ T cell trafficking is still limited. Leukocyte migration relies on vessels [51] composed of endothelial cells bound to each other by tight junction proteins and to the underlying matrix by integrin adhesion receptors [52, 53, 54]. Although epithelial barrier function demonstrated no changes from E2, the impact of E2 on *endothelial* barrier function and translocation of immune cells requires further study.

These results suggest no direct impact of E2 or progesterone on fibroblast function related to the production of extracellular matrix components. This data also demonstrates no impact of E2 on epithelial cells, at least in barrier function in the proximal airway, despite the presence of ER‐α receptors. Previous studies have demonstrated decreased expression of ER‐α, ER‐β, and PR in iSGS scar epithelium when compared to healthy mucosa. These findings have supported the idea that E2 may actually be protective in the proximal airway epithelial barrier function [15]. E2 has been shown to have similar effects in maintaining barrier function in mechanisms of cutaneous epidermal injury [55], colitis, and inflammatory bowel disease [56], as well as oral epithelial cells [57]. Our results demonstrate neither a protective nor detrimental effect of E2 on barrier function, as TEER levels did not significantly decrease or increase with exposure. The impact of female sex hormone on epithelial barrier function will require additional study; it is possible progesterone drives epithelial barrier dysfunction. Epithelial cells demonstrate higher levels of progesterone receptors on flow cytometry, but TEER assessments were only tested with E2. This is an area for future investigation.

Although TEER values were tested at a broad range of E2 exposure, this did not mimic fluctuations of hormone over time that are seen biologically. This is one limitation that will be an important factor to consider in future studies. Additional limitations of this study include the small sample size that may not capture disease heterogeneity and variation in severity that is seen clinically. The in vitro nature of this study limits its direct translatability to the complex, in vivo disease process. Additionally, the scope of this study focuses solely on the influence of estrogen in the development of subglottic stenosis. It does not address mechanisms of stenosis development in the absence of E2 responsiveness or outside the female population.

Even though negative results were obtained, our positive results suggest estrogen exposure increases angiogenesis, with a significant increase in endothelial branch length after E2 exposure. The combination of these negative and positive results has helped hone in on E2 impacting endothelial cells rather than fibroblasts or endothelial cells in iSGS. Although the role of E2 and endothelial cell alterations in the pathogenesis of iSGS requires further study, it provides new pharmacologic targets for treatment with the potential to repurpose readily available FDA‐approved drugs (i.e., selective ERb antagonist, Tamoxifen). Previous animal models have shown promise in the use of Tamoxifen for airway disease, specifically asthma models with a reduction of neutrophil infiltration of the airway [58, 59]. Alternatively, reagents targeting endothelial cell number or function (i.e., selective VEGF antagonist, Bevacizumab) might represent a new treatment strategy for fibrotic disease in the proximal airway.

## 5 Conclusion

Despite its relationship with disease phenotype and severity, the mechanistic role of E2 in subglottic fibrosis has not been well studied. In vitro experiments suggest E2 may act directly on endothelial cells to contribute to vascular remodeling in iSGS. E2 appears to have a limited direct impact on fibroblasts and epithelial cells in iSGS. Further studies are needed to elucidate how E2‐driven angiogenesis influences fibrosis in iSGS disease pathogenesis.

## Conflicts of Interest

The authors declare no conflicts of interest.

## Supporting information


**Table S1:** 48 genes associated with extracellular matrix.

## Data Availability

The data that support the findings of this study are available from the corresponding author upon reasonable request.
