# Supplementary material for: The Impact of Estrogen on Stromal Elements in the Proximal Airway in Idiopathic Subglottic Stenosis
Source: Laryngoscope. 2025 Nov 14;136(3):1349–56. doi: 10.1002/lary.70216 (PMC12913749; doi:10.1002/lary.70216)
Supplement: Supplementary file 1 — Table S1: 48 genes associated with extracellular matrix. [file LARY-136-1349-s001.docx]

Supplementary Table 1. 48 Genes Associated with Extracellular Matrix

| HGNC ID (gene) | Approved symbol | Approved name | Previous symbols | Aliases | Chromosome |
| --- | --- | --- | --- | --- | --- |
| HGNC:2197 | COL1A1 | collagen type I alpha 1 chain |  | OI4 | 17q21.33 |
| HGNC:2198 | COL1A2 | collagen type I alpha 2 chain | OI4 |  | 7q21.3 |
| HGNC:2200 | COL2A1 | collagen type II alpha 1 chain | SEDC, AOM | STL1 | 12q13.11 |
| HGNC:2201 | COL3A1 | collagen type III alpha 1 chain | EDS4A |  | 2q32.2 |
| HGNC:2202 | COL4A1 | collagen type IV alpha 1 chain |  |  | 13q34 |
| HGNC:2203 | COL4A2 | collagen type IV alpha 2 chain |  | FLJ22259, DKFZp686I14213 | 13q34 |
| HGNC:2204 | COL4A3 | collagen type IV alpha 3 chain |  |  | 2q36.3 |
| HGNC:2206 | COL4A4 | collagen type IV alpha 4 chain |  | CA44 | 2q36.3 |
| HGNC:2207 | COL4A5 | collagen type IV alpha 5 chain | ASLN, ATS |  | Xq22.3 |
| HGNC:2208 | COL4A6 | collagen type IV alpha 6 chain |  |  | Xq22.3 |
| HGNC:2209 | COL5A1 | collagen type V alpha 1 chain |  |  | 9q34.3 |
| HGNC:2210 | COL5A2 | collagen type V alpha 2 chain |  |  | 2q32.2 |
| HGNC:14864 | COL5A3 | collagen type V alpha 3 chain |  |  | 19p13.2 |
| HGNC:2211 | COL6A1 | collagen type VI alpha 1 chain |  |  | 21q22.3 |
| HGNC:2212 | COL6A2 | collagen type VI alpha 2 chain |  |  | 21q22.3 |
| HGNC:2213 | COL6A3 | collagen type VI alpha 3 chain |  |  | 2q37.3 |
| HGNC:33484 | COL6A4P1 | collagen type VI alpha 4 pseudogene 1 | DVWA | VWA6, DIVA, COL6A4, COL6A4P | 3p25.1 |
| HGNC:38501 | COL6A4P2 | collagen type VI alpha 4 pseudogene 2 |  | COL6A4 | 3q22.1 |
| HGNC:26674 | COL6A5 | collagen type VI alpha 5 chain | COL29A1 | FLJ35880, VWA4 | 3q22.1 |
| HGNC:27023 | COL6A6 | collagen type VI alpha 6 chain |  |  | 3q22.1 |
| HGNC:2214 | COL7A1 | collagen type VII alpha 1 chain | EBDCT, EBD1, EBR1 |  | 3p21.31 |
| HGNC:2215 | COL8A1 | collagen type VIII alpha 1 chain | C3orf7 | MGC9568 | 3q12.1 |
| HGNC:2216 | COL8A2 | collagen type VIII alpha 2 chain | FECD | PPCD, FECD1, PPCD2 | 1p34.3 |
| HGNC:2217 | COL9A1 | collagen type IX alpha 1 chain |  |  | 6q13 |
| HGNC:2218 | COL9A2 | collagen type IX alpha 2 chain | EDM2 | MED | 1p34.2 |
| HGNC:2219 | COL9A3 | collagen type IX alpha 3 chain |  | IDD, MED, EDM3, FLJ90759, DJ885L7.4.1 | 20q13.33 |
| HGNC:2185 | COL10A1 | collagen type X alpha 1 chain |  |  | 6q22.1 |
| HGNC:2186 | COL11A1 | collagen type XI alpha 1 chain | COLL6, DFNA37 | STL2, CO11A1 | 1p21.1 |
| HGNC:2187 | COL11A2 | collagen type XI alpha 2 chain | DFNA13, DFNB53 | HKE5 | 6p21.32 |
| HGNC:2188 | COL12A1 | collagen type XII alpha 1 chain | COL12A1L |  | 6q13-q14.1 |
| HGNC:2190 | COL13A1 | collagen type XIII alpha 1 chain |  |  | 10q22.1 |
| HGNC:2191 | COL14A1 | collagen type XIV alpha 1 chain | UND |  | 8q24.12 |
| HGNC:2192 | COL15A1 | collagen type XV alpha 1 chain |  |  | 9q22.33 |
| HGNC:2193 | COL16A1 | collagen type XVI alpha 1 chain |  |  | 1p35.2 |
| HGNC:2194 | COL17A1 | collagen type XVII alpha 1 chain | BPAG2 | BP180 | 10q25.1 |
| HGNC:2195 | COL18A1 | collagen type XVIII alpha 1 chain | KNO | KS, KNO1 | 21q22.3 |
| HGNC:2196 | COL19A1 | collagen type XIX alpha 1 chain |  |  | 6q13 |
| HGNC:14670 | COL20A1 | collagen type XX alpha 1 chain |  | KIAA1510 | 20q13.33 |
| HGNC:17025 | COL21A1 | collagen type XXI alpha 1 chain |  |  | 6p12.1 |
| HGNC:22989 | COL22A1 | collagen type XXII alpha 1 chain |  |  | 8q24.23-q24.3 |
| HGNC:22990 | COL23A1 | collagen type XXIII alpha 1 chain |  | DKFZp434K0621 | 5q35.3 |
| HGNC:20821 | COL24A1 | collagen type XXIV alpha 1 chain |  |  | 1p22.3 |
| HGNC:18603 | COL25A1 | collagen type XXV alpha 1 chain |  |  | 4q25 |
| HGNC:18038 | COL26A1 | collagen type XXVI alpha 1 chain | EMID2 | Emu2,EMI6 | 7q22.1 |
| HGNC:22986 | COL27A1 | collagen type XXVII alpha 1 chain |  | KIAA1870,MGC11337,FLJ11895 | 9q32 |
| HGNC:22442 | COL28A1 | collagen type XXVIII alpha 1 chain |  |  | 7p21.3 |
| HGNC:3778 | FN1 | fibronectin 1 |  |  | 2q35 |
| HGNC:17635 | CD274 | programmed cell death 1 ligand 1 | PDCD1LG1 |  | 9p24.1 |
